# WEE1 inhibition sensitizes osteosarcoma to radiotherapy

**DOI:** 10.1186/1471-2407-11-156

**Published:** 2011-04-29

**Authors:** Jantine PosthumaDeBoer, Thomas Würdinger, Harm CA Graat, Victor W van Beusechem, Marco N Helder, Barend J van Royen, Gertjan JL Kaspers

**Affiliations:** 1 Department of Orthopaedic Surgery, VU University Medical Center, PO box 7057, 1007 MB Amsterdam, the Netherlands; 2 Neuro-oncology Research Group, Departments of Neurosurgery and Paediatric Oncology/Haematology, VU University Medical Center, PO box 7057, 1007 MB Amsterdam, the Netherlands; 3 Molecular Neurogenetics Unit, Department of Neurology, Massachusetts General Hospital and Harvard Medical School, Boston, 13th Street, Building 149, Charlestown, MA, 02129 USA; 4 Department of Medical Oncology, RNA Interference Functional Oncogenomics Laboratory (RIFOL), VU University Medical Center, Amsterdam, the Netherlands; 5 Research institute MOVE/Skeletal Tissue Engineering Group Amsterdam (STEGA), PO box 7057, 1007 MB Amsterdam, the Netherlands; 6 Paediatric Oncology/Haematology, VU University Medical Center, PO box 7057, 1007 MB Amsterdam, the Netherlands

## Abstract

**Background:**

The use of radiotherapy in osteosarcoma (OS) is controversial due to its radioresistance. OS patients currently treated with radiotherapy generally are inoperable, have painful skeletal metastases, refuse surgery or have undergone an intralesional resection of the primary tumor. After irradiation-induced DNA damage, OS cells sustain a prolonged G_2 _cell cycle checkpoint arrest allowing DNA repair and evasion of cell death. Inhibition of WEE1 kinase leads to abrogation of the G_2 _arrest and could sensitize OS cells to irradiation induced cell death.

**Methods:**

WEE1 expression in OS was investigated by gene-expression data analysis and immunohistochemistry of tumor samples. WEE1 expression in OS cell lines and human osteoblasts was investigated by Western blot. The effect of WEE1 inhibition on the radiosensitivity of OS cells was assessed by cell viability and caspase activation analyses after combination treatment. The presence of DNA damage was visualized using immunofluorescence microscopy. Cell cycle effects were investigated by flow cytometry and WEE1 kinase regulation was analyzed by Western blot.

**Results:**

WEE1 expression is found in the majority of tested OS tissue samples. Small molecule drug PD0166285 inhibits WEE1 kinase activity. In the presence of WEE1-inhibitor, irradiated cells fail to repair their damaged DNA, and show higher levels of caspase activation. The inhibition of WEE1 effectively abrogates the irradiation-induced G_2 _arrest in OS cells, forcing the cells into premature, catastrophic mitosis, thus enhancing cell death after irradiation treatment.

**Conclusion:**

We show that PD0166285, a small molecule WEE1 kinase inhibitor, can abrogate the G_2 _checkpoint in OS cells, pushing them into mitotic catastrophe and thus sensitizing OS cells to irradiation-induced cell death. This suggests that WEE1 inhibition may be a promising strategy to enhance the radiotherapy effect in patients with OS.

## Background

Osteosarcoma (OS) is the most common primary malignant bone tumor in children and adolescents. The gold standard for treatment of OS consists of multi-agent neoadjuvant chemotherapy, radical excision of the tumor and adjuvant chemotherapy [1-3]. With this treatment regimen, 5-year survival rates of approximately 65% are obtained in localized disease. In patients with axial and/or inoperable OS, local control is difficult to achieve and there is a high risk of relapse and/or metastasis. The prognosis for these patients is worse with a 5-year survival of around 25% [4-7]. Clearly, alternative treatment options for OS are warranted for patients in whom local control can be scarcely achieved and therefore have a high risk of recurrence. Radiotherapy as a treatment modality for cancer has evolved over the past decades, but its use in OS treatment is controversial because OS is considered to be a relatively radioresistant tumor [2,3,5,7]. At present, radiotherapy is applied only in a select group of patients with OS, namely those who suffer from inoperable (advanced extremity, axial or head-and-neck) OS, patients with painful bone metastases and patients who refuse surgery. Radiotherapy can give local control in OS when applied as an adjuvant therapy in patients who have undergone an intralesional resection of the primary tumor with subsequent irradiation of the surgical margins [1-3,5,8-11]. Technical progression in the field of radiotherapy has facilitated a more precise localised delivery of radiation and thus warranted dose-intensification at the site of the tumor. This is of value since the high irradiation doses needed for tumor control are difficult to achieve in patients with tumors that lie in the proximity of delicate structures, as is often the case in axial OS. Regularly, adverse side effects limit the dose that can be applied. Although still considered an advanced technique, the use of proton radiotherapy can be even more exactly localized to deliver a higher irradiation dose in the tumor while sparing adjacent healthy tissues. The toxicity and efficacy of this method in bone sarcomas is studied in clinical trial setting [11,12]. Furthermore, the use of radiosensitizing drugs has further improved the anti-tumor efficacy of radiotherapy [3,5,8,13,14]. Conventional chemotherapy has been shown to enhance the effect of radiotherapy in OS. Gemcitabine (with or without Docetaxel) and Ifosfamide have been shown to be potent radiosensitizers [3,15]. Also, the use of 153-Samarium can enhance the anti-tumor effect of external beam radiotherapy in axial OS [3,5,9,13]. Thus, chemotherapeutic agents may be used as radiosensitizers in OS patients. Moreover, small molecule inhibitor drugs may serve as additional radiosensitizers [13,16].

**Figure 1:**
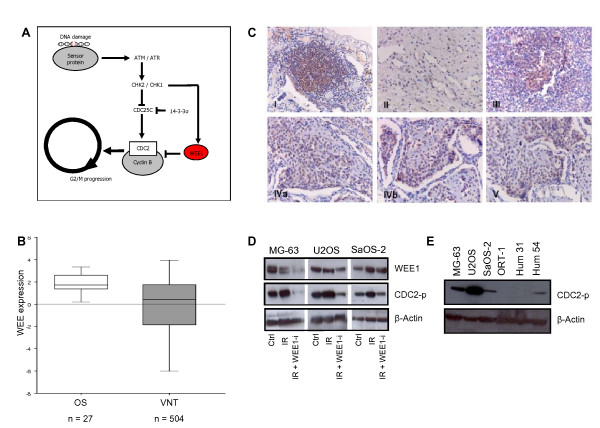
**WEE1 kinase regulates the onset of mitosis and is expressed in human OS**. (A) The Cyclin-B/CDC2 complex is considered the master switch for the G_2_/M transition and CDC2 is designated the general controller in the onset of mitosis. Inhibition of the Cyclin-B/CDC2 complex prevents mitotic entry. CDC2 activation is essential for G_2_/M transition and thus progression through the cell cycle and is regulated on multiple levels. Inhibitory phosphorylation of CDC2 is achieved by WEE1 and prevents proper association with Cyclin-B. Consequently, Cyclin-B and CDC2 cannot form a complex and the complex cannot be activated. This induces a G_2 _arrest and prevents the cell cycle to progress until DNA repair is completed. Dephosphorylation of CDC2 by the phosphatase CDC25C activates the Cyclin-B/CDC2 complex, allowing cell cycle progression. The activation status of the Cyclin-B/CDC2 complex is dependent on the balance between WEE1 kinase and CDC25C phosphatase activity, in which WEE1 kinase is the rate limiting, dominant molecule. (B) WEE1 mRNA expression is significantly higher in the OS samples when compared to the normal tissue samples (ANOVA: p < 0.0001). (C) Panel C shows immunohistochemical staining of sections of primary OS (extremity: II, III and axial: I, V) and OS lung metastasis (IVa,b). The brown nuclear staining indicates WEE1 expression in OS tumor tissue. (D) Western blot analysis of the effect of PD0166285 on WEE1 function. Irradiated (IR) cells show increased expression levels of CDC2-p^Y15 ^(CDC2-p) compared to untreated cells. After subsequent treatment with WEE1-inhibitor PD0166285 (WEE1-i), CDC2-p^Y15 ^expression levels are diminished, indicative of inhibition of WEE1 kinase activity. (E) Baseline expression levels of phosphorylated CDC (CDC2-p), the most important molecular target of WEE1 kinase, are distinctly lower in human primary osteoblasts compared to human OS cells.

Radiotherapy, like many other cancer treatments, induces damage to the DNA. Prolonged activation of cell cycle checkpoints (arrest) is one effective method exploited by cancer cells to repair DNA and thus evade apoptosis after DNA-damaging treatments [16-20]. When cells progress through the cell cycle despite the presence of DNA damage, as a result, they undergo a mitosis specific cell death programme called mitotic catastrophe [16-18,20-23]. Cancer cells often lack a functional G_0_/_1 _cell cycle checkpoint and therefore rely mainly on the G_2 _cell cycle arrest to gain time for DNA repair [20,23-27]. Therefore, one strategy to sensitize OS cells to DNA damaging treatments is to exploit their vulnerability in defective cell cycle regulation and prevent them from repairing the damaged DNA during G_2 _arrest. WEE1 kinase plays a dominant role in the sensitivity of cancer cells to DNA damage by inhibitory phosphorylation of Cyclin-Dependent-Kinase 1 (CDC2), thereby preventing mitotic entry, which is illustrated in Figure 1A, [16-20,27-33]. It has been shown that PD0166285, a small molecule WEE1 kinase inhibitor, can abrogate the G_2 _checkpoint in cancer cells, forcing DNA-damaged cells into premature mitotic entry thus inducing mitotic catastrophe and sensitizing the cells to apoptosis. The anti-tumor activity of WEE1 inhibition in combination with DNA damaging treatments has been demonstrated *in vitro *as well as *in vivo *models for different malignancies [16,21,28,29]. These promising preclinical results have led to the testing of a small molecule WEE1-inhibitor in a phase I clinical trial [27]. The aim of our study is to investigate if irradiation in combination with WEE1 inhibition could be used as a new therapeutic strategy to improve local control in the treatment of OS.

## Methods

### Cell culture, irradiation and compounds

Human osteosarcoma cell lines MG-63, U2OS and SaOS-2 were kindly provided by Dr. C. Löwik (Leiden University Medical Center, Leiden, the Netherlands), Dr. S. Lens (Dutch Cancer Institute, Amsterdam, the Netherlands) and Dr. F. van Valen (Westfalische Wilhelms-Universität, Münster, Germany) respectively. Human primary (short-term culture) osteoblasts (ORT-1, Hum31 and Hum54) were obtained from healthy patients undergoing total knee replacement after informed consent. Cells were cultured in D-MEM (Gibco, Invitrogen) supplemented with 10% fetal calf serum (FCS) and 1mg/mL Penicillin-Streptomycin (Gibco) at 37°C and 5% CO2 in a humidified incubator.

Cells were irradiated in a Gammacell^® ^220 Research Irradiator (MDS Nordion) at doses varying from 2 to 10 Gray (Gy). The WEE1-inhibitor PD0166285 (Pfizer, Ann Arbor, MI, USA) was diluted in PBS to the desired concentration of 0.5 μM.

### Immunohistochemistry

Paraffin embedded tissue samples of primary OS and OS lung metastases, obtained from excision specimens from our institute, were deparafinized and rehydrated. Endogenous peroxidase was inhibited by 30 minutes incubation of the sections in 0.3% H_2_O_2_, diluted in methanol. Antigens were retrieved by boiling in citrate buffer (pH 6) for 10 minutes, followed by successive rinses in phosphate-buffered saline (PBS) containing 0.5% Triton and then in PBS only. Slides were incubated for 10 minutes in 0.1 M glycine (diluted in PBS) and rinsed in PBS. Slides were incubated with mouse-anti-WEE1 (SantaCruz) O/N at 4°C. Visualisation was performed using the Power Vision^+ ^Poly-HRP IHC Kit (Immunologic) and tissue staining was performed with DAB chromogen solution. Slides were counterstained with hematoxylin, dehydrated and mounted. Placenta tissue served as positive control, prostate tissue served as negative control (not shown). Images were acquired at 20x objective.

### Western Blot

Basic expression levels of WEE1 and phosphorylated CDC2 in human OS cell lines and human primary osteoblasts were assessed by Western blot. Cells were lysed in phospho-lysis buffer containing Protease and Phosphatase Inhibitor Cocktails (Sigma). Proteins were quantified with the BCA protein Assay Kit (Pierce). A total of 40 μg protein was separated on a SDS-PAGE gel and transferred to a PVDF membrane, followed by incubation with the primary antibodies: mouse anti-WEE1 (SantaCruz), mouse anti-β-actin (SantaCruz) and rabbit anti-CDC2-p^Y15 ^(Abcam) and subsequently incubated with secondary goat-anti-mouse and goat-anti-rabbit immunoglobulins (DAKO). Protein detection and visualization was performed using ECL^+ ^Western Blotting Detection Reagents (Pierce).

Inhibition of WEE1 kinase activity and concomitant phosphorylation of CDC2 by the WEE1-inhibitor PD0166285 was also analyzed by Western blot analysis. Cells were plated and irradiated at a dose of 4 Gy in the presence or absence of 0.5 μM PD0166285. After 4 h treatment with 0.5 μM PD0166285, cells were lysed in phospho-lysis buffer, followed by Western blot analysis as described above.

### Cell Viability and apoptosis assay

For cell viability analysis, OS cells and primary osteoblasts were plated in 96-well format and irradiated at doses of 2, 3, 4, 6, 8 and 10 Gy. Cells were incubated with 0.5 μM PD0166285 or PBS directly post-irradiation. At 4 days (OS) and 9 days (osteoblasts) after treatment cell viability was assessed using the CellTiter-Blue Cell Viability Assay (Promega) according to the manufacturer's instructions.

To analyse apoptosis, OS cells were plated in white opaque 96-well plates and treated with 4 Gy irradiation or with combination treatment of 4 Gy and 0.5 μM PD0166285. At 6 h and 24h post-irradiation, caspase activity was measured using the Caspase-Glo 3/7 assay (Promega) according to the manufacturer's instructions.

Fluorescence and luminescence read-out was performed using a Tecan Infinite F200 Microplate Reader (Tecan Trading AG, Switzerland). Results were analysed using GraphPad Prism^® ^Version 5.01 (GraphPad Software, Inc. San Diego, CA, USA).

### Flow cytometry

Cell cycle distribution and the percentage of mitotic cells were analysed using flow cytometry. Cells were plated and treated with 4 Gy irradiation, 0.5 μM PD0166285 or combination treatment. At 20 h after treatment cells were trypsinized, washed in PBS containing 1% FCS and fixed in 70% ice-cold ethanol for 24 h. After fixation, cells were washed with PBS containing 1% FCS and incubated with rat-anti-phospho-histone H3 (PHH3) antibody (BD Pharmingen) in PBS containing 1% BSA for 2 h at room temperature, followed by secondary antibody incubation with rabbit-anti-rat/FITC immunoglobulins (DAKO) in PBS containing 1% BSA for 30 minutes at room temperature in the dark. Cells were washed once and DNA was stained with 50 μg/mL propidium iodide (PI) solution in the presence of 250 μg/mL RNAseA (Sigma). The DNA content and the percentage of PHH3 positive cells were measured using a FacsCalibur Flow Cytometer and the Cell Quest Pro programme (Becton Dickinson) and results were subsequently analysed using ModFitLT software (Verity Software House, Topsham, ME, USA).

### Immunofluorescent Staining

OS cells were seeded on glass coverslips in 24-well plates and treated with 4 Gy irradiation or with combination treatment of 4 Gy and 0.5 μM PD0166285. At 1 h and 24 h post-irradiation cells were fixed in 2% paraformaldehyde. Prior to staining, the cells were rinsed in PBS and permeabilized in PBS containing 0.1% Trition X-100 for 30 minutes at room temperature and blocked in PBS containing 5% FCS. Slips were incubated with mouse-anti-γ-histone-H2AX (Millipore) in PBS containing 5% FCS O/N at 4°C, followed by secondary antibody incubation rabbit-anti-mouse/FITC immunoglobulins (DAKO) in PBS containing 5% FCS for 30 minutes at room temperature in the dark. Slips were rinsed in PBS thrice and nuclei were stained with DAPI (1:10 000) in PBS at room temperature in the dark, followed by successive rinses in PBS and sterile water. The slips were then mounted on glass slides, fixed with Mowiol and analyzed with a Carl Zeiss Axioskop 20 microscope at 100x objective.

## Results

To investigate whether WEE1 could be a suitable drug target in human OS we first explored its expression levels. From publicly available gene expression data in the GEO Expression Omnibus (http://www.ncbi.nlm.nih.gov/geo: GSE14827), we analyzed WEE1 expression in 27 OS samples and 504 various normal tissue samples using the software programme R2 [34]. We determined that WEE1 kinase is overexpressed in OS compared to various normal tissues, as shown in Figure 1B. When comparing the mRNA expression level of WEE1 in OS samples to the normal various tissue samples, one-way analysis-of-variance (ANOVA) shows that WEE1 expression is significantly higher in the OS samples (p < 0.0001). In addition, we determined WEE1 protein expression in human OS tissue sections by immunohistochemical staining. Five out of 6 tested tumors had positive nuclear WEE1 staining (Figure 1C). The nuclear localization of the protein is in concordance with its role in cell cycle regulation. These data indicate that WEE1 is indeed expressed by OS and could thus serve as a potential drug target.

Next, we assessed whether PD0166285 can inhibit WEE1 kinase function by determining phosphorylation of its target CDC2 (resulting in CDC2-p^Y15^) using Western blot analysis. Irradiated cells showed a moderate increase in WEE1 expression and a more profound increase in expression of CDC2 -p^Y15 ^compared to untreated cells (Figure 1D). This supports the notion that WEE1 kinase plays a role in the response to DNA damage by phosphorylation of CDC2. Subsequent treatment with PD0166285 diminished the expression of CDC2 -p^Y15 ^after irradiation. This shows that PD0166285 effectively inhibits WEE1 activity and thus reduces the inhibitory phosphorylation of CDC2 in OS cells.

To analyse how baseline WEE1 and CDC2- p^Y15 ^levels in OS cells compare to normal cells, we included a western blot analysis. Figure 1E shows that CDC2- p^Y15 ^levels in human primary osteoblasts are negligible in comparison to the OS cell lines. WEE1 expression in the osteoblasts could not be visualised.

**Figure 2:**
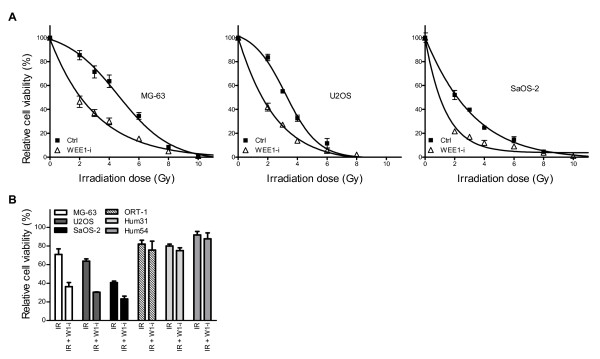
**WEE1-inhibition sensitizes OS cells to γ-irradiation but not normal cells**. (A) Indicated are dose response curves of irradiated cells (squares) and cells treated with IR + WEE1-inhibitor (triangles). The curves represent three experiments, performed in triplicate; error bars indicate standard deviation (SD). The sensitizing effect of WEE1 inhibition is significant in all three OS cell lines (student's t-test: p < 0.01) (B) Analysis of cell viability of human OS cell lines and human primary osteoblasts treated with 4 Gray (Gy) IR in the presence or absence of WEE1-inhibitor. Bars represent experiments performed in triplicate; error bars indicate SD. The OS cell lines MG-63, U2OS and SaOS-2 show a 2-fold decrease in cell viability when treated with combination therapy, whereas human primary osteoblasts ORT-1, Hum31 and Hum54 do not show sensitization to radiotherapy in the presence of the WEE1-inhibitor.

To investigate the effects of WEE1 inhibition on OS cell survival after γ-irradiation-induced DNA damage, we compared cell viability in irradiated cells in the presence or absence of the WEE1-inhibitor PD0166285. Figure 2A shows that WEE1 inhibition using PD0166285 at a non-toxic dose (0.5 μM) increased cell death after 2 to 6 Gy γ-irradiation in the OS cell lines MG-63, U2OS and SaOS-2 (p < 0.01), whereas treatment with 0.5 μM WEE1-inhibitor alone showed no effect on cell viability (data not shown). To ascertain that WEE1 inhibition does not radiosensitize normal cells, we compared cell viability of human primary osteoblasts to osteosarcoma cell lines after 4 Gy irradiation, in the presence or absence of 0.5 μM PD0166285. Figure 2B shows that in the osteosarcoma cell lines there is a clear sensitization to irradiation treatment, with approximately a 2-fold reduction in cell viability after combination treatment. In contrast, in the human osteoblasts no such effects were seen. There is a minor decrease in cell viability due to the irradiation treatment, but WEE1 inhibition does not enhance cell death. The results were consistent for all three tested human primary osteoblasts. From this we conclude that OS cells are indeed sensitized to irradiation whereas normal cells are not.

**Figure 3:**
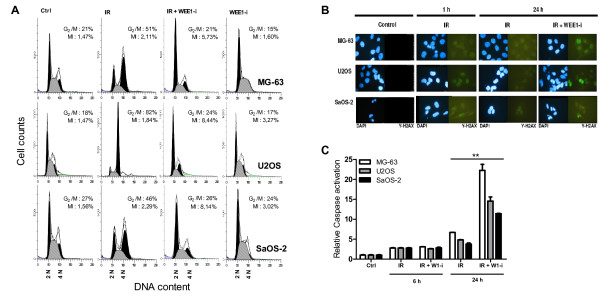
**WEE1 inhibition abrogates the γ-irradiation induced G_2_/M cell cycle arrest in OS cells and leads to mitotic catastrophe**. (A) Histograms of FACS cell cycle analysis of OS cells treated with 4 Gy IR, 0.5 μM WEE1-inhibitor, and combination treatment. Percentages of cells in G_2_/M phase and the mitotic index (MI) in percentages are shown. After irradiation, the percentages of cells in G_2_/M phase increased, whereas the percentages of mitotic cells remained unaltered. After subsequent treatment with WEE1-inhibitor, the G_2 _arrest is completely abrogated; the percentages of cells in G_2_/M phase return to control values, while the percentages of mitotic cells increase dramatically. This indicates a forced progression through the G_2 _cell cycle checkpoint into mitosis. (B) Fluorescence microscopy images of nuclei (blue) with ionizing radiation induced foci (IRIF) (green) indicating DNA damage, visualized using immunofluorescent γ-histone-H2AX staining of DNA breaks at 1 h and 24 h post-irradiation. Cells treated with IR alone show fewer IRIF at 24 h after treatment than cells treated with IR + WEE1-inhibitor. (C) Caspase activation in OS cells treated with 4 Gy IR or 4 Gy IR + 0.5 μM WEE1-inhibitor 6 h and 24 h after treatment. Bars represent experiments performed in triplicate; error bars indicate SD. After 6 h, there is a mild induction of caspase activity. Caspase activation levels are comparable between the two treatment groups. At the 24 h time point there is a significantly higher caspase activation in the cells treated with combination treatment for all cell lines (student's t-test: p < 0.01).

To investigate if the sensitizing effect of WEE1 inhibition in OS could be explained by mitotic catastrophe, we looked into three aspects of this phenomenon. We performed FACS cell cycle analysis of cells treated with 4 Gy γ-irradiation, 0.5 μM PD0166285, and combination treatment. Cells were stained with PI to analyse DNA content and with PHH3 to distinguish the fraction of mitotic cells from the cells in G_2_/M phase. Treatment with the WEE1-inhibitor alone did not alter the cell cycle distribution (Figure 3A). Irradiation of the cells resulted in arrest in the G_2_/M phase, indicated by an accumulation of cells with 4N DNA content, but a stable percentage of mitotic cells. However, upon treatment of the irradiated cells with the WEE1-inhibitor, a clear abrogation of G_2 _arrest was observed. Additionally, there was a 2 to 4-fold increase in the percentage of mitotic cells.

To assess the extent of γ-irradiation-induced double strand DNA breaks (DSBs), we visualized the number of ionizing radiation induced foci (IRIF) with DSB marker γ-H2AX at 1 h and 24 h after irradiation, in cells irradiated at a dose of 4 Gy in the presence or absence of 0.5 μM PD0166285. Figure 3B shows that DNA damage is visible at 1 h after irradiation. In the irradiated cells, only a few residual foci are detectable after 24h compared to the 1h time point, indicating that DNA repair has occurred or is still ongoing. The shape of the nuclei is regular and there are no clear signs of apoptosis. In contrast, the cells treated with irradiation in combination with WEE1-inhibitor show extensive remaining DNA damage after 24 h with irregularity and fragmentation of nuclei indicative of nuclear envelope disassembly and apoptosis. From this we derive that in WEE1 inhibited cells DNA repair is not effectively realized.

To verify that cell death occurs as a result of apoptosis we analysed caspase activation in irradiated cells in the presence or absence of WEE1-inhibitor (Figure 3C). At 6 h post-irradiation there is a mild caspase activation in cells treated with irradiation alone or with combination treatment. However, at 24 h post-irradiation there is a distinct difference in caspase activation between irradiated cells (4 to 6-fold) and cells treated with the combination of irradiation and WEE1-inhibitor (11 to 22-fold) (p < 0.01 for all three cell lines). Taken together, this implies that cells treated with the WEE1-inhibitor are forced to proceed through the G_2 _cell cycle checkpoint into mitotic entry despite the presence of DNA damage and are therefore sensitized to γ-irradiation-induced apoptosis.

## Discussion

In this work, we explore the possibility to use WEE1 inhibition as a new therapeutic strategy in OS. The use of WEE1-inhibitor PD0166285 to obtain radiosensitization in various malignancies has been reported previously [18,21,28,29]. The radiosensitization effect is described to be particularly effective in, if not limited to, p53 deficient malignancies [18,28]. Interestingly, we have found that our tested cell lines can all be sensitized to irradiation, regardless of their p53 status (wt in U2OS, mutated in MG-63 and null in SaOS-2). This, we ascribe to the idea that a defective G_1 _checkpoint is not necessarily caused by p53 mutations alone but rather a disruption in the p53 pathway, which can be caused by other aberrations within this pathway. We show that after irradiation, OS cells accumulate in a predominant G_2 _arrest, the abrogation of which effectively leads to mitotic catastrophe.

As was reported previously [21,27], our results confirm that normal cells remain unaffected by WEE1 inhibition after irradiation. We tested human primary osteoblasts for their response to irradiation in the presence or absence of WEE1-inhibitor. While there was a minor effect of irradiation on cell viability, no radiosensitization by PD0166285 was observed. This is likely explained by a functional G_1 _checkpoint with concurrent wild type p53 expression. This indicates that WEE1 inhibition is a safe strategy to apply in OS patients because the radiosensitization would be cancer cell specific.

Apart from being a regulator of mitotic entry, WEE1 has been described to also affect other important cellular processes, such as regulation of mitotic spindle formation, positioning and integrity, microtubule stabilization and heat shock protein 90 (Hsp90) phosphorylation [29,35,36]. In this paper, we have not examined these phenomena, but it could be that the disruption of one of these processes contributes to the observed phenotype. It may be interesting to study these additional effects in the future.

Timing of combination therapy is important to obtain optimal treatment efficacy. It was reported that CDC2 is transiently phosphorylated to induce an arrest at the G_2_/M checkpoint for 12 h after irradiation treatment and that DNA damage could be repaired in 12-24 h after irradiation [19]. Our results support this; in irradiated cells, we observed only few remaining foci of DNA damage after 24h, whereas cells treated with irradiation and WEE1-inhibitor had many residual foci after 24h, indicating that they were unable to perform DNA repair. This suggests that DNA damaged cells are especially susceptible to WEE1-inhibitor in the first 12h after induction of DNA damage. In our experimental set-up, the cells were treated with WEE1-inhibitor directly after irradiation and show a good sensitization. This suggests that cells do not have to be arrested in G_2_/M phase to be susceptible to WEE1 inhibition, but rather that the inability to activate (or maintain) the G_2 _checkpoint in the presence of DNA damage leads to sensitization. In *in vivo *testing of WEE1-inhibitors, different approaches have been applied. Mir et al. [21] administered WEE1-inhibitor at 5 consecutive days around the irradiation dose, whereas Hirai et al. [18] first administered DNA damaging agents, followed by WEE1-inhibitor after a 24 hour interval. Both groups showed enhanced anti-tumor efficacy. What will be the most optimal schedule for radiotherapy combined with WEE1 inhibition in OS remains to be tested *in vivo*.

## Conclusion

Radiotherapy is a controversial topic in the treatment of OS. Its efficacy is limited in this cancer and therefore it is not widely applied. Novel small molecules, in particular WEE1-inhibitor drugs may serve as radiosensitizers in OS. WEE1 kinase is expressed in OS and plays a critical role in DNA repair by maintaining the G_2 _cell cycle arrest through inhibitory phosphorylation of CDC2. Our results show that the WEE1-inhibitor PD0166285 can abrogate the DNA damage induced G_2_/M cell cycle arrest in OS cells, forcing the cells into mitotic catastrophe and thus causing radiosensitization. WEE1 could therefore be a strategic, cancer cell specific drug target and its inhibition could be an effective strategy to enhance the efficacy of radiotherapy in OS.

## Abbreviations

DSB: Double strand break; CDC2: Cyclin Dependent Kinase 1; Gy: Gray; γ-H2AX: {Gamma}-Histone H2AX; Hsp90: Heat shock protein 90; IR: Irradiation; IRIF: ionizing radiation induced foci; Inh: Inhibitor; MI: Mitotic index; OS: Osteosarcoma; PBS: Phosphate Buffered Saline; PD: Pro-drug; PHH3: Phospho-Histone H3; SD: Standard Deviation.

## Competing interests

The authors declare that they have no competing interests.

## Authors' contributions

JP performed the experiments, interpreted the experimental data, conceived and drafted the manuscript. TW and HG conceived the study and experiments. TW, HG, VB and MH helped interpret the experimental data and corrected the manuscript. BR reviewed and corrected the manuscript. GK supervised the project and critically reviewed the contents of the manuscript. All authors read and approved the final manuscript.

## Pre-publication history

The pre-publication history for this paper can be accessed here:

http://www.biomedcentral.com/1471-2407/11/156/prepub
